# Novel roles of vibration transmittance in fracture testing

**DOI:** 10.1186/s13019-019-0884-0

**Published:** 2019-03-12

**Authors:** Aaron R. Casha, Kieran Chircop, Marilyn Gauci, Joseph N. Grima

**Affiliations:** 1 Department of Cardiothoracic Surgery, Mater Dei Hospital, Msida, Malta; 2 Faculty of Medicine, University of Malta, Msida, Malta; 3 Department of Medical Imaging, Mater Dei Hospital, Msida, Malta; 4 Metamaterials Unit, Faculty of Science, University of Malta, Msida, Malta

**Keywords:** Sternotomy, Vibration transmittance, Dehiscence, Ultrasound

## Abstract

This letter re-assesses a publication in the Journal of Cardiothoracic Surgery entitled ‘Vibration transmittance measures sternotomy stability – a preliminary study in human cadavers.’ The roles of ultrasound in testing for sternotomy stability and that of stress vibration transmittance in cases of fracture of the posterior table of the sternum or in hairline undisplaced fractures are examined in view of their differing sound wave frequency ranges.

## Main text

We read with interest the article by Hautalahti et al. about the application of vibration transmittance to measure sternotomy stability and congratulate them on a new direction in basic research in sternotomy testing [1]. We believe that with future developments, this technique may become particularly useful in cases where only the posterior table of the sternum is fractured or in hairline undisplaced fractures where ultrasound may give a false negative result. The technique may be further enhanced by a Valsalva manoeuvre to increase intrathoracic pressure or by upper limb movements to apply micro displacement.

**Fig. 1:**
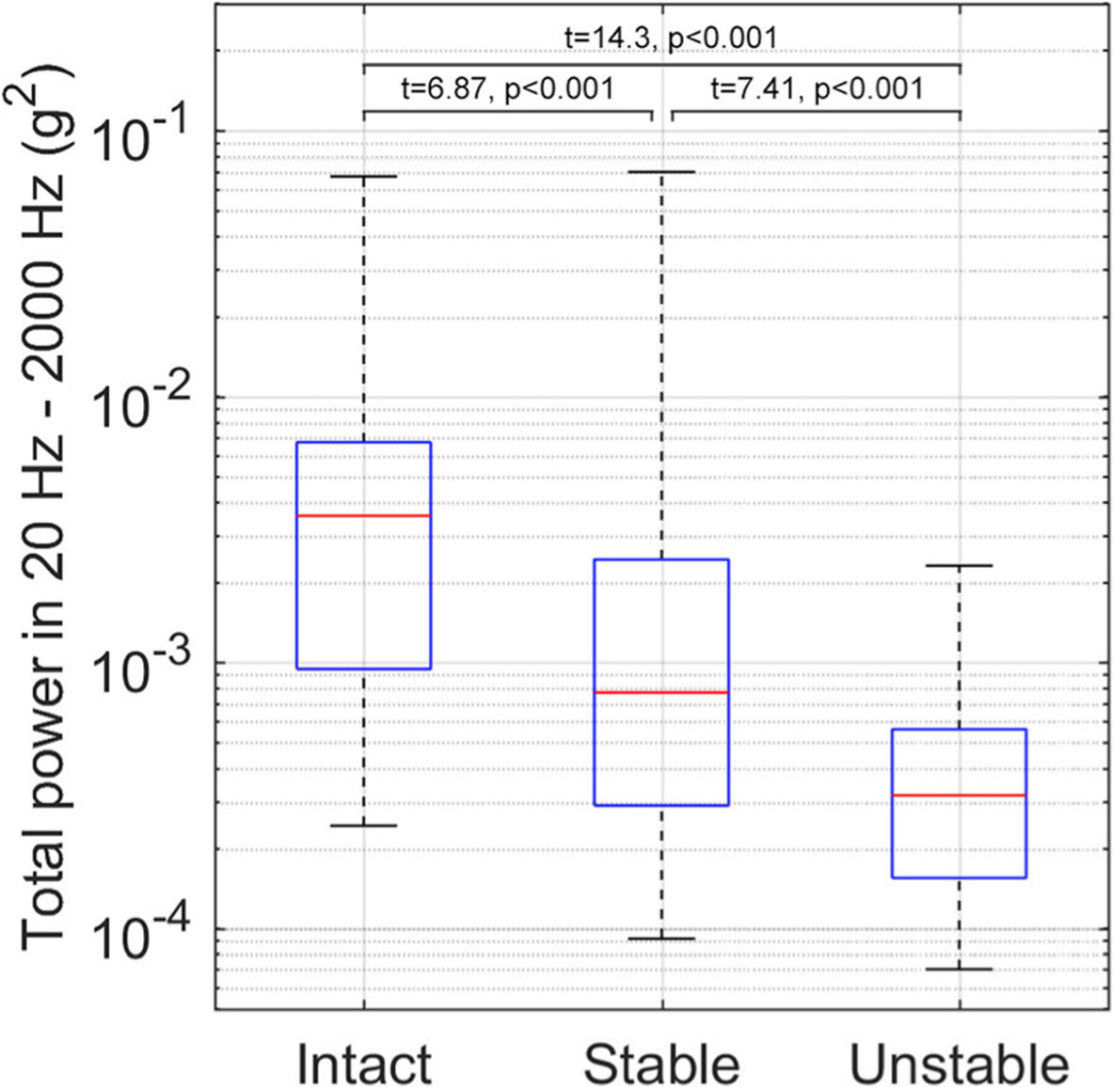
Combined results of vibration transmittance testing at the 2nd – 4th rib levels of intact sternums, stable fixation with 6 steel wires and unstable fixation with a 10 mm dehiscence gap. Taken from Hautalahti et al. [1]

However, we equally believe that the technique as presented still needs further developments before it can be considered as an alternative to current protocols. For example, we note that whilst vibration transmittance testing showed statistical significance between intact, stable wired and unstable sternums, there was a big overlap between stable and unstable at 10 mm gap, see Fig. 1 [1]. This suggests that there are cases that may not be easily discernible using this technique. At sternal gaps smaller than 10 mm, distinguishing between the stable and unstable sternum is likely to become even more of an issue.

Ultrasound permits visualisation of cortical discontinuity due to the highly reflective bone–soft tissue interface as well as identification of haematomas and pleural effusions and has a sensitivity and specificity of 100% in sternal fractures [2]. Post-sternotomy ultrasound can accurately assess gap measurements, detect small changes in sternal separation and measure micro-motion in an acute surgical population, permitting serial monitoring with a minimal detectable change of 0.14 mm in both lateral and antero–posterior planes [3, 4]. Though Hautalahti et al. contend that ultrasound carries a risk of wound contamination in the early post-operative period, this is easily obviated by using sterile transducer covers and gel [5].

We hope vibration transmittance will be further developed. Nevertheless until such developments are made from this preliminary study, we consider that the visualisation and measurement accuracy of ultrasound, together with its widespread availability within hospitals and primary care, makes ultrasound the current investigation of choice in the context of sternotomy instability testing post-surgery, features that vibration transmittance lacks at its present stage of development.

## Conclusions

Vibration transmittance testing and ultrasound both use sound waves although at different frequency ranges, with 20 kHz as the threshold separating the frequency bands. However vibration transmittance relies on transmission through the medium, while ultrasound depends on reflection and has difficulty penetrating bone. This means that ultrasound cannot detect posterior sternal table or hairline undisplaced fractures, which can be detected by vibration transmittance. Thus we believe that ultrasound and vibration transmittance can become complimentary techniques in the future.
